# Effect of radiation timing on the capsular contracture of implant-based breast reconstruction: A retrospective cohort study

**DOI:** 10.1097/MD.0000000000041438

**Published:** 2025-02-07

**Authors:** Yoshiko Iwahira, Gojiro Nakagami, Kojiro Morita, Hiromi Sanada

**Affiliations:** a Breast Surgery Clinic, Tokyo, Japan; b Department of Gerontological Nursing/Wound Care Management, Graduate School of Medicine, The University of Tokyo, Tokyo, Japan; c Global Nursing Research Center, Graduate School of Medicine, The University of Tokyo, Tokyo, Japan; d Ishikawa Prefectural Nursing University, Ishikawa, Japan.

**Keywords:** complication, expander, implant, Implant-based breast reconstruction, radiation

## Abstract

Breast reconstruction using tissue expanders and silicone implants has become increasingly popular in Japan since health insurance began covering the procedure in 2013. Radiotherapy after mastectomy is recommended for certain patients and has been identified as a major risk factor for capsular contractures. However, the effect of radiation therapy timing on the development of capsular contracture has not yet been thoroughly studied. This study aimed to evaluate the effects of radiation therapy timing on the development of capsular contractures in patients who underwent implant-based breast reconstruction performed by a single expert plastic surgeon. This retrospective cohort study included 341 patients undergoing implant-based breast reconstruction and irradiation between April 2003 and March 2019. Patients were categorized based on radiation and operation types. Variables included postirradiation skin condition, implant mobility, and expander placement position. The outcome measure was the development of capsular contracture, assessed using the Baker Classification. Of 340 patients, 43 developed severe capsular contracture (Baker classification Grade III) within 2 years (cumulative incidence, 12.6 %). No significant relationship was found between the radiation or operation type and capsular contracture. Instead, postirradiation skin redness, implant mobility, skin pinchability after 1 year, and expander positioning were found to be significant factors affecting capsular contracture development. The timing of radiation therapy was not a determinant of capsular contracture development. Factors such as postirradiation skin inflammation, implant mobility, ability to pinch the skin, and expander position play pivotal roles in determining capsular contracture development.

## 1. Introduction

Breast reconstruction surgery employing tissue expanders and silicone implants has historically been the predominant choice, given its minimal invasiveness and superior aesthetic outcomes.^[1]^ The number of patients undergoing implant-based breast reconstruction is increasing in Japan because health insurance has covered operation fees since 2013.^[2]^ Radiation therapy after mastectomy has been recommended by the guidelines for breast cancer treatment for patients with tumor size greater ≥ 5 cm, breast cancer with 4 positive nodes, or locally recurrent tumors.^[3]^

Several types of irradiation treatment orders may affect aesthetic results and the occurrence of complications or failures; however, only a few studies have investigated the effect of radiation timing on aesthetic results after implant-based breast reconstruction. A chart review of patients who underwent immediate 2-stage expander/implant with/without postmastectomy radiation therapy demonstrated no significant difference between the postmastectomy radiation to expanders group and postmastectomy radiation to implants group after expander exchange in the development of capsular contracture.^[4]^ Another retrospective matched-cohort study reported that a history of radiation did not affect the outcomes of implant-based breast reconstruction in patients with Hodgkin lymphoma.^[5]^ Andersen et al, reported that no significant difference in the overall rate of major or minor complications were observed between patients receiving radiotherapy on tissue expander or implant.^[6]^ Yoon et al, also reported the timing of radiation therapy (before or after implant exchange) did not have a significant impact on complication risks or most patient-reported outcomes.^[7]^ In an observational study by Nava et al, compared to patients who underwent implant-based breast reconstruction but did not undergo irradiation, there were no significant differences in the development rate of severe capsular contracture between postmastectomy radiotherapy timings (during tissue expansion vs permanent implant).^[8]^ On the other hand, Cordeiro et al, reported that in the group that received radiation after implant exchange, the incidence of Baker classification Grade III or IV capsular contracture was 44.6%, which was higher compared to 15.9% in the group that received radiation to the tissue expander.^[9]^ In a recent meta-analysis investigating the optimal timing of postmastectomy radiotherapy in 2-stage implant-based breast reconstruction, the authors compared outcomes between patients receiving radiation on the tissue expander vs those receiving radiation on the permanent implant after exchange. The analysis, which included 8 studies and 899 cases, found that the risk of severe capsular contracture was significantly lower in patients who received radiation to the tissue expander, however, the authors highlighted the need for further studies due to the low level of evidence and insufficient sample sizes.^[10]^ Taken together, as seen in a survey of reconstructive practices among American plastic surgeons in pre- and postmastectomy radiation, there is little consensus on the ideal type and timing of reconstruction in pre- and postoperative radiation.^[11]^

As previous studies have included data from multiple centers or even single centers with data from multiple plastic surgeons’ procedures, it has been difficult to accurately assess the impact of radiation therapy timing on the development of capsular contracture owing to the variability in the quality of breast reconstructive procedures. Therefore, this study examined the timing of operations and radiation therapy each year and evaluated its effect on the development of capsular contracture in patients who underwent breast reconstructive surgery performed by a single expert plastic surgeon.

## 2. Patients and methods

### 2.1. Study design and patient

This retrospective cohort study included patients who presented to a clinic where implant-based breast reconstruction was performed by a single expert plastic surgeon between April 2003 and March 2019, and who had been continuously attending the clinic for at least 2 years after implant-based breast reconstruction surgery. Patients who had undergone bilateral implant-based breast reconstruction were excluded (N = 1). Clinical guidelines recommend radiation after mastectomy if there are ≥ 4 metastases in the axillary lymph nodes. Radiation may also be administered if recurrence occurs after mastectomy or in cases of positive margins.

### 2.2. Exposure variable: implant-based breast reconstruction and radiation order

Radiation was classified into 4 types according to the timing of the implant-based breast reconstruction surgery. Type 1 (mastectomy → radiation → expander insertion) involves delayed reconstruction. Type 2 (partial mastectomy → radiation → recurrence → mastectomy → expander insertion → mastectomy → expander placement) is mastectomy due to intramammary recurrence after post-conservative surgery radiation. The expander may be inserted either immediately or after a delay; however, in both cases, the patient has already undergone radiation therapy. Therefore, it is necessary to check if the skin can be pinched before inserting the expander. If the skin is too hard to pinch, the expander will not expand, and there is a high possibility of capsular contracture and extrusion after silicone replacement. Types 3 and 4 involved immediate procedures. Type 3 (mastectomy = expander placement → radiation → implant exchange) involved irradiation during skin expansion, and type 4 (mastectomy = expander placement → implant exchange → radiation) involved irradiation after implant replacement.

The type of operation was classified as either immediate or delayed. Type 1 (postmastectomy radiation) involves delayed reconstruction. Type 3 (radiation during expansion) and type 4 (radiation after implant exchange) were observed immediately. Type 2 (radiation after partial mastectomy) was sub-classified as immediate and delayed according to the timing of the expander placement. Proportions of radiation types and operation types were summarized per year to assess yearly trends. All patients followed the guidelines for breast cancer treatment. Radiation was delivered using a linear accelerator at 4 to 6 MV of energy, with total radiation dose of 50 Gy, delivered in 25 fractions of 200 cGy each over 5 weeks.

### 2.3. Other variables

To evaluate the condition of the skin immediately after irradiation, redness and thinning of the skin were assessed. We also checked whether the mobility of implants was maintained and whether the skin could be pinched 1 year after irradiation.

Redness: The degree of redness and heat of the skin was evaluated visually by an experienced medical professional. Redness was rated as present when there was obvious redness and heat compared to the surrounding skin and skin of the contralateral breast.

Thinning: Skin thinning was evaluated based on palpation and visual observation. An experienced evaluator examined the thickness of the skin by touch and deemed thinning present if there was obvious melting or loss of subcutaneous tissue or fat (i.e., papery, ribbed, or telangiectasia).

Mobility: This is an indicator of the ease of skin movement and flexibility and was evaluated by palpation. The evaluator gently shook or pulled the skin to check its ease of movement and deemed mobility to be absent if movement was poor.

Skin pinchability: The evaluator assessed whether the skin could be pinched with a thumb and forefinger.

Because our previous study showed that misalignment of the expander predicts the development of capsular contracture, we evaluated whether the expander was in the normal anatomic position.^[12]^ The position of the expander was classified into 1 of the following 6 categories based on the inferior margin of the healthy breast: symmetry, 1 lateral finger upward, 2 lateral fingers upward, 1 lateral finger downward, 2 lateral fingers downward, and outside. Lateral finger method is a simple and commonly used clinical method where a finger is tilted sideways to measure how many fingers it corresponds to in width. This technique is often employed as a quick reference for gauging size or distance, making it convenient for use in the clinical setting. Symmetry was deemed the normal position.

## 3. Outcome measures

The degree of capsular contracture was assessed by a plastic surgeon according to the Baker classification.^[13]^ The severity of capsular contracture was assessed 2 years after implant placement, and an unfavorable outcome was considered grade III or IV capsular contracture according to the previous reports, but there were no cases of grade IV in the present study.

### 3.1. Statistical analysis

The number of patients was summarized according to the implant year for the operation and radiation types to describe the time-course change. Cross-tabulation was used to compare the frequencies before and after 2013 using the chi-squared test. The operation type and skin/implant status according to radiation type are summarized. Patients were assigned into 2 groups based on the occurrence of capsular contracture, and attributes were compared between the groups using a t-test or chi-squared test. To examine whether operation, radiation types, and other skin/implant statuses after radiation affect the development of capsular contracture, we used multivariate logistic regression analysis and adjusted for confounding variables to estimate odds ratio. All statistical analyses were conducted using STATA/MP17 (STATA Corp., College Station).

### 3.2. Ethical considerations

Since this study used reviewed medical chart data as a retrospective observational study, written informed consent from the participants was unnecessary. An announcement regarding the study and the option for participants to opt out was posted on the website of our clinic. The study protocol was approved by the Ethics Committee of the Breast Surgery Clinic, Tokyo, Japan, which included external reviewers. This study was conducted according to the principles of the Declaration of Helsinki.

## 4. Results

**Figure 1.**
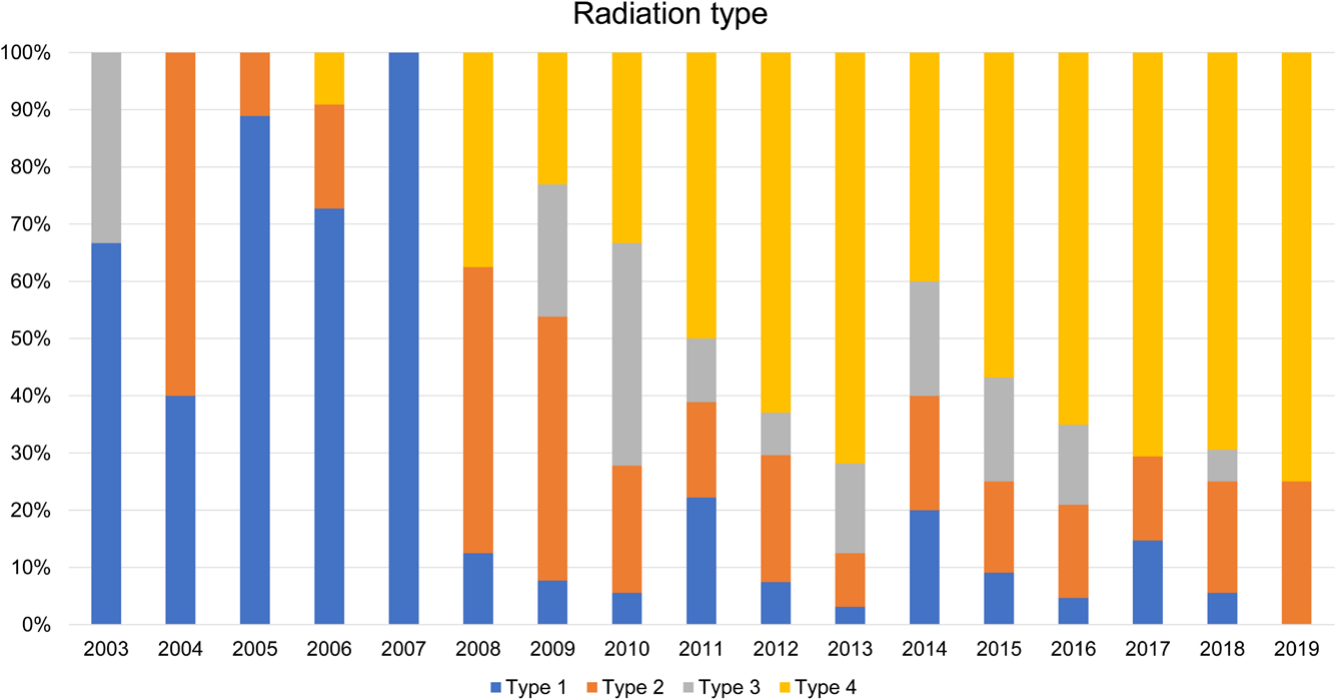
Trends in radiation types from 2003 to 2019.

**Figure 2.**
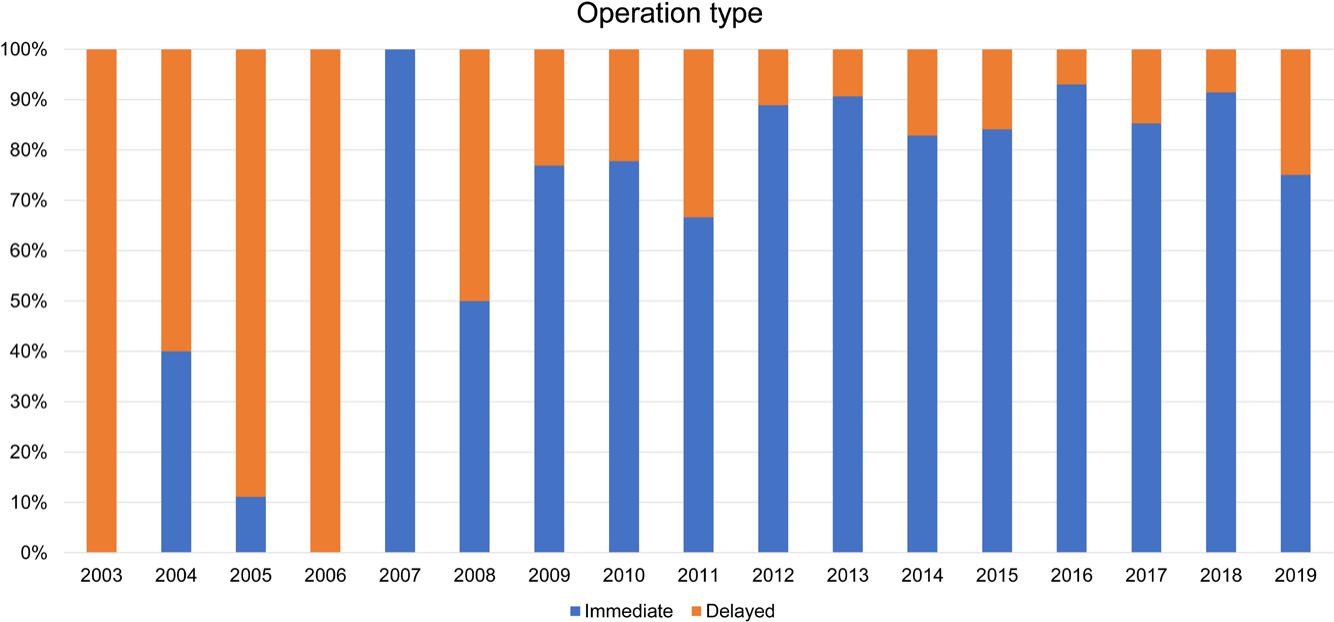
Trends in operation types from 2003 to 2019.

From April 2003 to March 2019, 340 patients undergoing implant-based breast reconstruction and irradiation were retrospectively included in this study. The recruitment summary is as follows: type 1 (N = 51), type 2 (N = 65), type 3 (N = 43), and type 4 (N = 181) for radiation types and immediate operation (N = 267) and delayed operation (N = 73) for operation types. Figure 1 illustrates the trends in radiation and operational types from 2003 to 2019. The delayed operation was the most common type of operation until 2006; however, since 2013, immediate operation comprised over 80% of all operations (Fig. 2). For radiation type, type 1 accounted for most cases until 2007, but type 4 has been gradually increasing since 2008 and has accounted for most cases since 2013, except for 2014 (Fig. 1). Following the initiation of insurance coverage for implant-based breast reconstruction surgery in Japan in 2013, there was a significant difference in both radiation (Table 1) and operation types (Table 2) (*P* < .001).

**Table 1:** Radiation types before 2012 and after 2013.

	Type 1		Type 2		Type 3		Type 4		Total		*P*
	(N = 51)		(N = 65)		(N = 43)		(N = 181)		(N = 340)		
Before 2012	30	(26.5)	29	(25.7)	15	(13.3)	39	(34.5)	113	(100.0)	<.001
After 2013	21	(9.3)	36	(15.9)	28	(12.3)	142	(62.6)	227	(100.0)	

Note: The Chi-squared test was used.

**Table 2:** Operation types before 2012 and after 2013.

	Immediate		Delayed		Total		*P*
	(N = 267)		(N = 73)		(N = 340)		
Before 2012	68	(60.2)	45	(39.8)	113	(100.0)	<.001
After 2013	199	(87.7)	28	(12.3)	227	(100.0)	

Note: The Chi-squared test was used.

Table 3 shows the operation type and skin/implant status according to radiation type. The frequency of skin redness and thinning immediately after radiation and implant mobility 1 year after radiation were significantly different among the 4 radiation types (*P* < .001, each).

**Table 3:** Surgical type and skin/implant status according to the radiation type.

		Type 1		Type 2		Type 3		Type 4		Total		*P*
Operation type	Immediate	5	(9.8)	51	(78.5)	41	(95.3)	170	(93.9)	267	(78.5)	<.001
	Delayed	46	(90.2)	14	(21.5)	2	(4.7)	11	(6.1)	73	(21.5)	
Skin redness (immediately after radiation)	Absent	50	(98.0)	64	(98.5)	35	(81.4)	136	(75.1)	285	(83.8)	<.001
	Present	1	(2.0)	1	(1.5)	8	(18.6)	45	(24.9)	55	(16.2)	
Skin thinning (immediately after radiation)	Absent	39	(76.5)	62	(95.4)	42	(97.7)	179	(98.9)	322	(94.7)	<.001
	Present	12	(23.5)	3	(4.6)	1	(2.3)	2	(1.1)	18	(5.3)	
Implant mobility (1 yr after radiation)	Absent	17	(33.3)	14	(21.5)	16	(37.2)	94	(51.9)	141	(41.5)	<.001
	Present	34	(66.7)	51	(78.5)	27	(62.8)	87	(48.1)	199	(58.5)	
Skin pinchability (1 year after radiation)	Absent	4	(7.8)	2	(3.0)	6	(14.0)	26	(14.4)	38	(11.2)	.070
	Present	47	(92.2)	63	(97.0)	37	(86.0)	155	(85.6)	302	(88.8)	
Expander location	Normal	37	(72.6)	49	(75.4)	31	(72.1)	150	(82.9)	267	(78.5)	.208
	Malposition	14	(27.5)	16	(24.6)	12	(27.9)	31	(17.1)	73	(21.5)	

Note: The Chi-squared test was used.

Of the 340 patients, 43 developed severe capsular contracture within 2 years (cumulative incidence, 12.6%). Table 4 shows patient characteristics according to capsular contracture development. Multiple logistic regression analysis showed no significant relationship between operation/radiation type and capsular contracture. Skin redness immediately after radiation (OR = 3.00), implant mobility (OR = 0.13), skin pinchability (OR = 0.35) 1 year after radiation, and expander location (OR = 4.49) were significantly associated with capsular contracture (Table 5).

**Table 4:** Patient characteristics by capsular contracture.

		Without capsular contracture	With capsular contracture	*P*
		N = 297	N = 43	
Age		44.6 (7.9)	45 (8.5)	.850
Operation type	Immediate	229 (77.1)	38 (88.4)	.110
	Delayed	68 (22.9)	5 (11.6)	
Radiation type	Type 1	47 (15.8)	4 (9.3)	.054
	Type 2	62 (20.9)	3 (7.0)	
	Type 3	36 (12.1)	7 (16.3)	
	Type 4	152 (51.2)	29 (67.4)	
Skin redness (immediately after radiation)	Absent	256 (86.2)	29 (67.4)	.004
	Present	41 (13.8)	14 (32.6)	
Skin thinning (immediately after radiation)	Absent	282 (94.9)	40 (93.0)	.490
	Present	15 (5.1)	3 (7.0)	
Implant mobility (1 yr after radiation)	Absent	104 (35.0)	37 (86.0)	<.001
	Present	193 (65.0)	6 (14.0)	
Skin pinchability (1 yr after radiation)	Absent	22 (7.4)	16 (37.2)	<.001
	Present	275 (92.6)	27 (62.8)	
Expander location	Normal	242 (81.5)	25 (58.1)	.001
	Malposition	55 (18.5)	18 (41.9)	

Note: N (%) or mean (standard deviation). The chi-square test or *t*-test were used.

**Table 5:** Risk factors for severe capsular contracture.

Variables	Odds ratio	95% confidence intervals		*P*
		Lower	Upper	
Age	1.01	0.97	1.06	.580
Operation type				
Immediate	Reference			
Delayed	0.36	0.05	2.62	.310
Radiation type				
Type I	Reference			
Type II	0.49	0.06	3.99	.506
Type III	1.16	0.13	10.01	.893
Type IV	1.10	0.15	8.01	.927
Skin redness (immediately after radiation)	3.00	1.24	7.26	.015
Skin thinning (immediately after radiation)	5.03	0.69	36.52	.110
Implant mobility (1 yr after radiation)	0.13	0.05	0.34	<.001
Skin pinchability (1 yr after radiation)	0.35	0.15	0.84	.018
Expander location (Malposition)	4.49	1.94	10.37	<.001

Note: Logistic regression was used.

## 5. Discussion

This retrospective cohort analysis from a single institution, overseen by a distinguished plastic surgeon with roughly 2 decades of experience, aimed to determine the role of radiation therapy timing in the onset of capsular contracture. Contrary to the initial presumptions, the study revealed that neither the timing of radiation therapy nor the operation type was significantly associated with the incidence of capsular contracture. In contrast, variables such as immediate postoperative skin redness, implant motility, skin pinchability after 1 year, and malpositioning of the expander were identified as pivotal factors influencing the development of capsular contracture.

The change in the number of irradiated cases indicated a history of breast cancer surgery in Japan. The number of patients who underwent conservative surgery and number of patients who were irradiated increased, but insurance coverage for immediate surgery led to a decrease in conservative surgery and a decrease in irradiation. Initially, there were many delayed surgery (type 1) cases, and the number of conservative surgeries increased; however, there were many complications due to irradiation in type 2 cases in which intraductal breast recurrence or appearance problems led to total mastectomy. While autologous reconstruction is generally considered safer after radiation therapy, there are many patients, such as those with Type I or Type II, who do not wish to undergo autologous reconstruction. Some may have a history of abdominal surgery, be unable to commit to a long hospital stay, or prefer to avoid scarring on other parts of their body. Additionally, in Japan, where the culture of bathing is important, there are many patients who prefer alternatives to autologous reconstruction for these reasons. Accordingly, breast surgeons have begun to recommend total mastectomy and implant-based breast reconstruction, which have exploded with insurance coverage.

Radiation is a major risk factor for capsular contracture.^[14]^ For example, a prospective study in Denmark that included 717 delayed breast implant reconstruction procedures demonstrated that radiation could increase the risk of capsular contracture.^[15]^ A study investigating the pathogenesis of radiation-induced capsular contracture indicated the importance of the wingless signaling pathway for fibroproliferation associated with capsular contracture in expander/implant breast reconstruction.^[16]^ Our previous study histopathologically compared irradiated and nonirradiated breast skin and demonstrated 5 characteristic changes: hyperplasia of the epidermis, flattening of the papillary layer, atrophy of dermal appendages, high density of dermal collagen fibers, and unidirectional alignment of dermal collagen fibers.^[17]^ A study investigating gene expression patterns using biopsies from irradiated and healthy nonirradiated capsular tissue harvested during implant exchange following immediate breast reconstruction surgery found specific inflammatory responses.^[18]^

Immediate expander placement is beneficial for patients; however, treatment prior to mastectomy is not completely predictable and may interfere with reconstructive procedures, especially those involving expanders/implants. Breast cancer practice guidelines recommend radiation therapy after mastectomy for both tumors > 5 cm in diameter and breast cancer with 4 positive nodes.^[3]^ Consequently, radiation therapy after expander implantation is increasing. Patients who have completed chemotherapy prior to mastectomy require radiation therapy during expander implantation. Since 2013, more cases have been treated with mastectomy first, allowing chemotherapy to be administered during expander implantation. One problem with immediate surgery is that adjuvant therapy has not been determined preoperatively. If preoperative chemotherapy is administered as in type 3, irradiation during extension is preferable if irradiation becomes necessary. If chemotherapy has not yet been administered, as in type 4, chemotherapy is administered after insertion of the expander, and irradiation is performed after replacement.

The cumulative incidence of Baker classification Grade III capsular contractures was 12.6% in the study cohort. In Nava study, ≥Grade III capsular contracture developed within a median follow-up period of 50 months in 56.9% and 32.0% of patients receiving radiotherapy during permanent implantation or tissue expansion, respectively.^[8]^ In Cagli observation, when radiation is applied to the implant, there is a higher chance of developing severe capsular contracture; however the incidence rate is much higher than that of the present study, it is difficult to generalize the result to our cohort.^[19]^ Those huge differences may be attributed to differences in follow-up periods (assessment timing), breast shape and volume, body mass index, and operation skills. In that study, replacement surgery and subsequent patient monitoring were performed.

Emphasis was placed on 3 evaluation criteria: postirradiation skin color alterations, ability of skin pinching, and implant movement. A higher prevalence of capsular contracture was observed in patients who exhibited reddened and inflamed skin immediately after irradiation. This aligns with prior observations suggesting pronounced skin inflammation as a precursor to capsular contracture, underscoring the necessity of surgical precision to mitigate skin tension and the resultant inflammation.^[20–22]^ Furthermore, echoing the findings from our previous research, misalignment of the expander emerged as a significant risk factor for radiotherapy. Maintenance of implant mobility and the ability to pinch the skin 1 year post-operatively served as indicators of the efficacy of post-reconstructive self-care, including skin maintenance. For skin care, twice-daily massage with a moisturizing cream containing oil (squalane) can be recommended. Thorough patient education is paramount, and consistent self-care practices are important.

### 5.1. Limitations of the study

Given that numerous postradiation patients cease follow-up visits a year post-replacement and that recurrences and mortality are prevalent, conclusions derived from this study’s cohort might not be generalizable to patients who experience recurrence. Furthermore, while this study’s exclusive focus on patients treated by a single plastic surgeon at 1 institution allows for the consideration of unmeasured confounding variables, particularly concerning the surgical technique, its findings might not readily extend to patients from diverse clinical settings. We acknowledge the limitations of this retrospective study, including the inability to apply standardized scoring systems such as LENT-SOMA due to the lack of detailed data in the medical records. Moving forward, we plan to conduct prospective studies incorporating the LENT-SOMA scoring system to provide a more comprehensive evaluation of radiation-induced skin toxicity.^[23]^

## 6. Conclusion

The development of severe capsular contracture of postirradiation reconstruction was determined not by the timing of radiation therapy but by the postirradiation skin inflammatory response, implant mobility, skin pinchability, and expander position.

## Acknowledgments

The authors appreciate all participants of this study.

## Author contributions

**Conceptualization:** Yoshiko Iwahira, Gojiro Nakagami, Hiromi Sanada.

**Data curation:** Yoshiko Iwahira.

**Formal analysis:** Yoshiko Iwahira, Gojiro Nakagami, Kojiro Morita.

**Investigation:** Yoshiko Iwahira.

**Methodology:** Gojiro Nakagami, Kojiro Morita, Hiromi Sanada.

**Supervision:** Hiromi Sanada.

**Visualization:** Kojiro Morita.

**Writing – review & editing:** Yoshiko Iwahira, Hiromi Sanada.

**Writing – original draft:** Gojiro Nakagami, Kojiro Morita.
